# Difficulty of predicting lymph node metastasis on CT in patients with rectal neuroendocrine tumors

**DOI:** 10.1371/journal.pone.0211675

**Published:** 2019-02-11

**Authors:** Hajime Ushigome, Yosuke Fukunaga, Toshiya Nagasaki, Takashi Akiyoshi, Tsuyoshi Konishi, Yoshiya Fujimoto, Satoshi Nagayama, Masashi Ueno

**Affiliations:** Department of Gastroenterological Surgery, Gastroenterological Center, Cancer Institute Hospital, Japanese Foundation for Cancer Research, Koto-ku, Tokyo, Japan; Osaka Medical Center for Cancer and Cardiovascular Diseases, JAPAN

## Abstract

**Background:**

Surgical indications for rectal neuroendocrine tumors with potential lymph node metastasis remain controversial. Although accurate preoperative diagnosis of nodal status may be helpful for treatment strategy, scant data about clinical values of lymph node size have been reported. The aim of this retrospective study was to investigate the relationship between lymph node size and lymph node metastasis.

**Methods:**

Participants comprised 102 patients who underwent rectal resection with total mesenteric excision or tumor-specific mesenteric excision and in some cases additional lateral pelvic lymph node dissection for rectal neuroendocrine tumor between June 2005 and September 2016. All lymph nodes from specimens were checked and measured.

**Results:**

Pathological lymph node metastasis was confirmed in 37 patients (36%), including 6 patients (5.8%) with lateral pelvic lymph node metastasis. A total of 1169 lymph nodes in the mesorectum were retrieved from all specimens, with 78 lymph nodes (6.7%) showing metastasis. Mean length (long-axis diameter) of metastatic lymph nodes in the mesorectum was 4.31 mm, significantly larger than that of non-metastatic lymph nodes (2.39 mm, P<0.01). The optimal cut-off of major axis length for predicting mesorectal lymph node metastasis was 3 mm. We could predict lymph node metastasis in only 7 patients (21%) from preoperative multidetector-row computed tomography.

**Conclusions:**

Metastatic lymph nodes were small, so predicting lymph node metastasis from preoperative computed tomography is difficult. Alternative modalities with a scan width less than 3 mm may be needed to predict lymph node metastasis of rectal NET with low cost and labour requirements.

## Introduction

Rectal neuroendocrine tumour (NET) is relatively rare disease. The annual incidence of this pathology is reportedly 0.14–0.76 cases per 100 000 population, with particularly high prevalence among Asian/Pacific Islanders, Native Americans, and African Americans [1]. This has also been reported and recognized as a pathological manifestation of mild-to-moderate nuclear atypia and localized disease, mostly with very small rates of metastasis to regional lymph nodes (LNs) or distant organs [2]. However, because of similar cancer-specific survival rates of rectal NET with regional or distant metastasis of rectal adenocarcinoma [3–5] according to some recent reports of long-term follow-up data, rectal NET is now considered a malignant disease [1,6]. As difficulty with chemotherapy has been reported [7–9], not only local resection but surgical resection with LN dissection around the rectum if LNs metastases are suspected must be used to achieve cure.

National Comprehensive Cancer Network guidelines recommend radical resection with LN dissection for rectal NET >2 cm in diameter [10]. The European Neuroendocrine Tumor Society and North American Neuroendocrine Tumor Society recommend radical resection with LN dissection for rectal NET >2 cm in size and for 1 to 2cm tumors with muscular invasion or positive nodes [11–13]. However, several reports have described tumor size, depth of invasion and presence of lymphovascular invasion as important factors influencing LN metastasis [4,5,14,15]. In 2010, Chino et al., from our institution, advocated more precise criteria for radical operation with total mesenteric excision (TME) or tumor-specific mesenteric excision (TSME) [16], meaning the inclusion of LN dissection around the rectum [17]. Focusing on LN metastasis, we noticed that metastatic LNs from rectal NET seemed rather small when referring to papers about surgical outcomes after laparoscopic rectal excision with LN dissection using the above criteria [5]. Globally, computed tomography (CT), especially images using a scan width of 5mm, is generally considered a routine modality to determine operative indications and/or preoperative staging. Our observation regarding the small size of metastatic LNs in rectal NET raises the clinical question regarding whether CT is a reasonable method to evaluate metastatic LN disease. We therefore designed this retrospective study to investigate the size of LNs collected from patients with rectal NET who underwent TME or TSME at our institution, to confirm the clinical value of LN size in rectal NET.

## Materials and methods

### Study population

This retrospective study involved human specimens or tissues, and was approved by the institutional review board of the Cancer Institute Hospital (Ariake, Tokyo; approval number “2017–1148”). Written informed consent was waived because this was retrospective design and the date were analyzed anonymously.

This was a single-center, retrospective study involving 102 patients who underwent rectal resection with TME or TSME for rectal NET between June 2005 and September 2016 at Cancer Institute Hospital, Japanese Foundation for Cancer Research, Tokyo, Japan. Demographic and pathological features were obtained from the database of the Cancer Institute Hospital. Tumors were pathologically diagnosed as rectal carcinoid and classified according to the criteria of the World Health Organization and Union for International Cancer Control, 7th edition [1]. Surgical treatment with radical LN dissection and rectal resection was performed based on the following criteria: maximum tumor diameter >10 mm; tumors with suspected presence of LN metastasis or invasion to the muscle layer as determined by endoscopic ultrasonography, computed tomography (CT), or in some cases magnetic resonance imaging (MRI); and positive resection margins, presence of lymphovascular invasion or NET Grade 2 [17] on pathological examination of the specimen after endoscopic mucosal/submucosal resection (ER) (n = 66, 64%) and transanal local resection (TAR) (n = 2, 1.9%) or biopsy. In all cases, additional surgery following previous local resection (with TAR or ER) was performed after a minimum interval of 2 months.

### Surgical procedures

Rectal resection with TME or TSME was performed by laparoscopic surgery in 97 cases and by open surgery in 5 cases. For both techniques, patients were treated under general anaesthesia in a lithotomy position. Laparoscopic surgery was performed under pneumoperitoneum with carbon dioxide gas insufflation via a 5 port approach, as reported previously from our institution [5]. The inferior mesenteric artery was ligated at the level of the origin or just peripheral to the left colic artery, depending on the circumstances of the patient, including physical characteristics and preoperative LN metastasis. For cases with lateral pelvic LN (LPLN) swelling suspected to represent metastasis based on a longitudinal diameter >7 mm, LPLN dissection (LPLD) was performed, again as reported from our institution [18]. Decisions relating to the operation were made at multidisciplinary team conferences according to the preoperative criteria of LN metastasis with longitudinal diameter >8 mm in the mesorectum or >7 mm in the lateral pelvic region.

### Lymph nodes

LNs in surgical specimens were identified and isolated by surgeons after surgery, and the numbers and distributions were recorded. After formalin fixation, more than two pathologists cut LNs along the long axis and performed microscopic examinations of haematoxylin- and eosin- stained specimens. Retrospectively, surgeons checked all LNs on saline-coated glass slides and measured the long axes of each node every 1 mm under microscopy and with the naked eye. We recorded LN lengths of 0–1.4999 mm as 1 mm, and lengths over 10 mm as >10 mm. While LNs in the mesorectum were investigated in those patients who underwent rectal resection with mesorectal excision, LPLNs were also investigated in those who underwent the above rectal surgery plus lateral LN dissection. However, only LNs in the mesorectal region were targeted for calculating sensitivity and specificity because not all cases in this series underwent lateral LN dissection.

### Statistical analysis

Metastatic LNs were evaluated by receiver operating characteristic (ROC) curves using the area under the curve (AUC) to determine the optimal cut-off value. Continuous variables were compared using the Wilcoxon signed-rank test. Survival outcomes were determined using the Kaplan-Meier method. Values of p < 0.05 were considered statistically significant. Statistical analyses were performed using JMP version 8.0.2 software (SAS Institute, Cary, NC).

## Results

### Patient demographic and pathological characteristics

The demographic and pathological characteristics of patients are provided in S1 Table and are summarized in Table 1. All distant metastases were detected in the liver. Mean size of the primary tumor with positive LNs was 12.6mm, and tumor grade with LN metastasis was G1 in 76% (25/33) and G2 in 24% (8/33) of patients.

**Table 1:** Patient demographic and pathological characteristics.

Characteristics	All cases (n = 102)
Age (median)	53 (30–76)
Sex (male:female)	64:38
Location	
upper	5
middle	37
lower	60
Tumor size (median)	8 (3–35)
0–10 mm	56
≧10 mm	46
Multiple tumors	4
Preoperative treatment	
endoscopic resection	66
transanal resection	2
Tumor depth	
submucosa	91
mp∼	11
Laparoscopic surgery	97
LPLD	7
Number of lymph nodes (median)	11 (1–27)
pN0/pN1	65/37
LPLN (+)	6
Lymphatic invasion (+)	45
unknown	3
Venous invasion (+)	57
unknown	6
Grade(G1/G2)	75/18
unknown	9
pStage I/IIA/IIB/IIIA/IIIB/IV	62/2/1/0/34/3
Distant metastasis	3
Recurrence	3

※LPLN lateral pelvic lymph node dissection

### Size and distribution of mesorectal LNs

**Fig 1:**
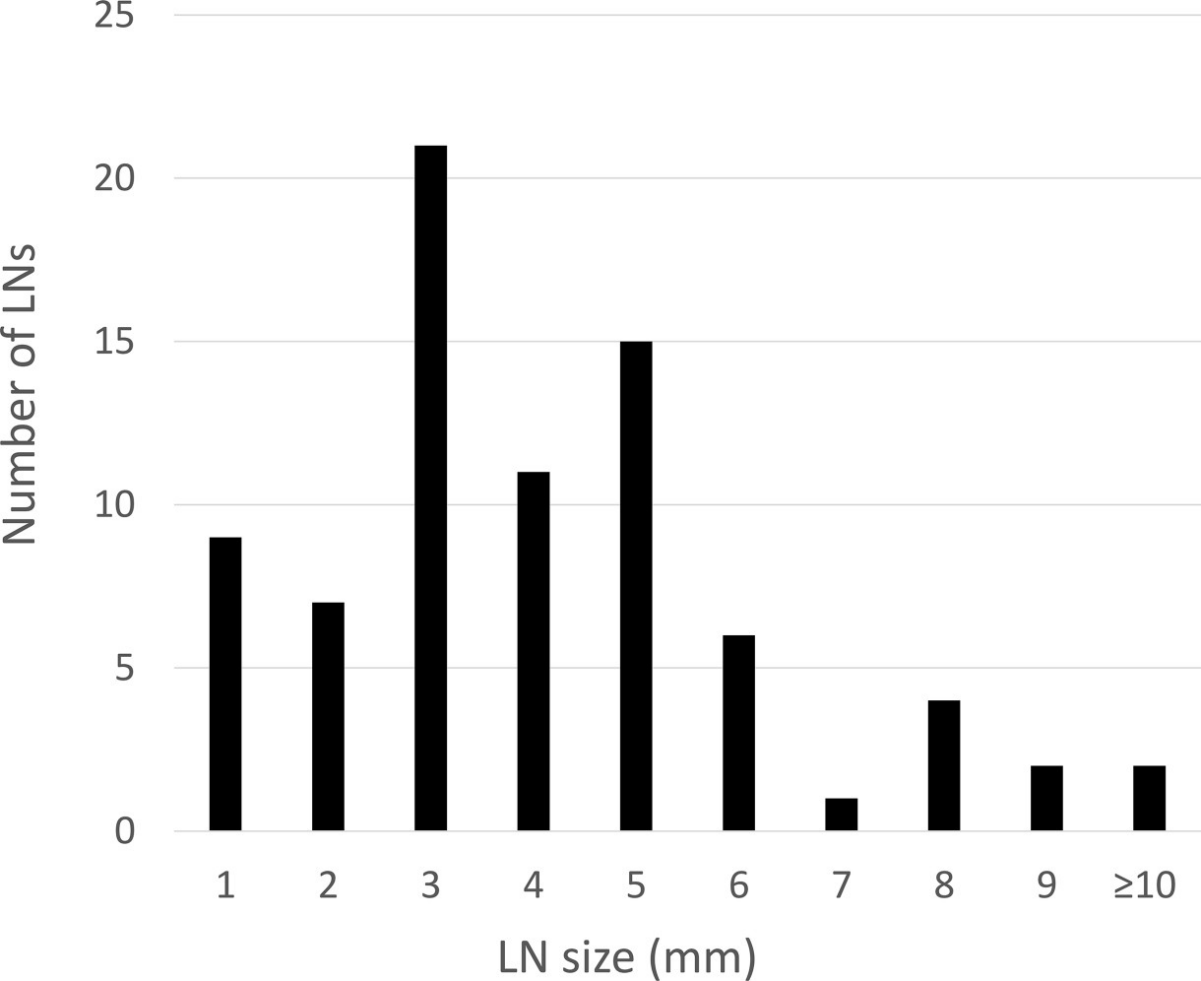
Size and distribution of mesorectal metastatic LNs.

**Fig 2:**
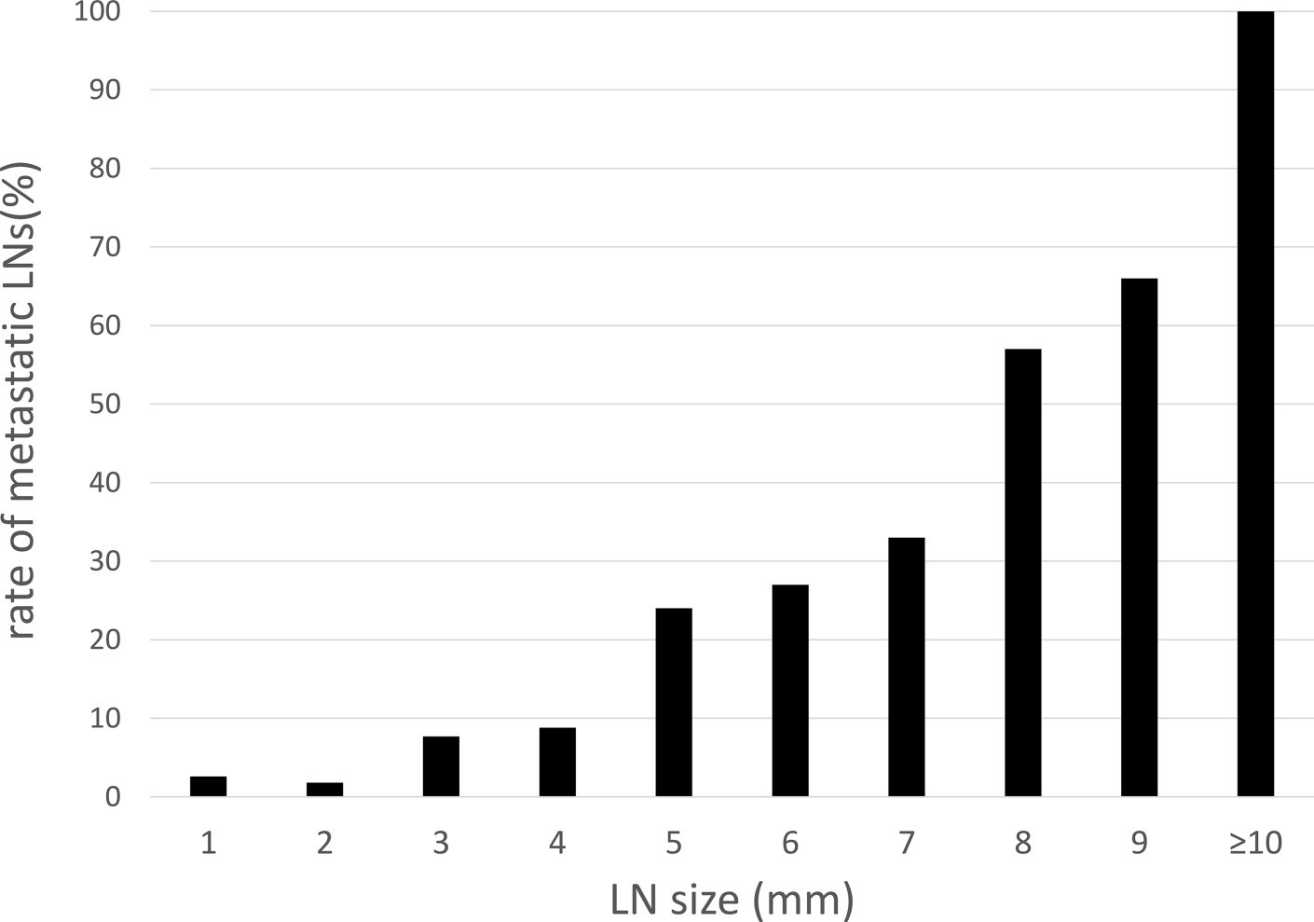
Rate of metastatic LNs according to size of mesorectal LNs.

A total of 1169 mesorectal LNs (n = 102) were retrieved from all specimens. Among those, 78 LNs (6.7%) showed metastasis. Mean long-axis diameter of retrieved LNs was 2.52 mm (range 1–28 mm), with metastatic LNs (4.31 mm; range, 1–28 mm) significantly larger than non-metastatic LNs (2.39 mm; range, 1–8 mm, p<0.01) S2 Table. In the metastatic LN group, 37 LNs (47%) measured <3 mm, 63 LNs (81%) were ≤5 mm, and only 8 LNs (10%) were >8 mm (Fig 1). Metastasis-positive rates for each length of LN are shown in (Fig 2). Almost all LNs were located around the superior rectal artery (95%), with the remaining (5%) around the inferior mesenteric artery S3 Table.

### Lateral pelvic lymph nodes

LPLD was performed for 7 patients who had presented with LPLN swelling >7 mm in size on preoperative CT or MRI. One patient displayed simultaneous liver metastasis. Metastatic LPLNs were confirmed in 6 patients (86%), representing the only LPLN metastases in 4 of the 6 patients (66%). Sixteen LPLNs were retrieved from specimens, of which six showed metastasis. These positive LNs had a mean size of 11.6 mm (range, 7–18 mm) S4 Table. In the 6 patients with metastatic LPLN, one showed local recurrence and one showed distant recurrence.

### Optimal cut-off values for predicting mesorectal lymph node metastasis

**Fig 3:**
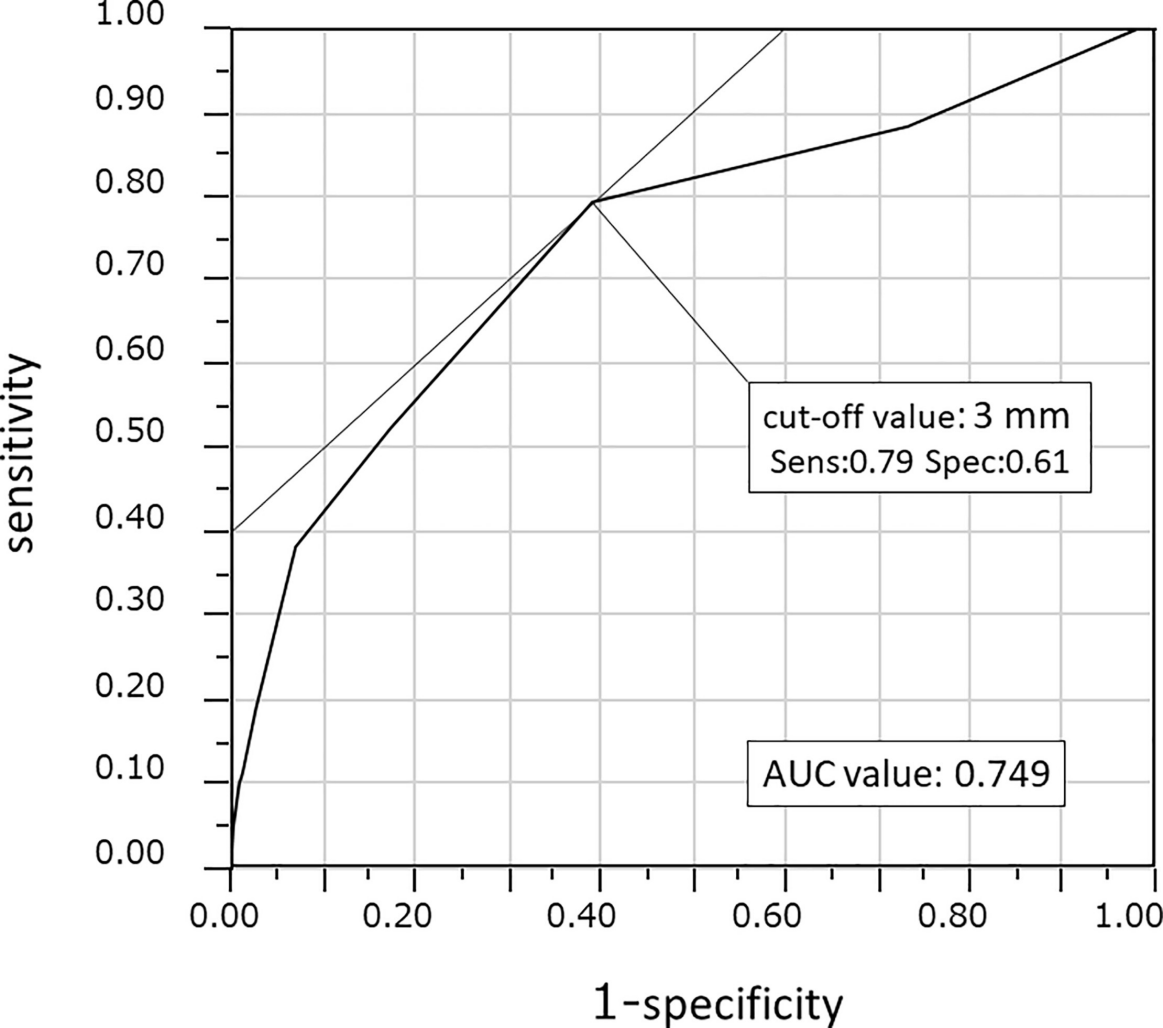
ROC curve showing optimal cut-off value of 3 mm (sensitivity, 0.795; specificity, 0.61).

Optimal diagnosis of positive LNs on preoperative examination requires determination of suitable LN length criteria. ROC curves were generated and the AUC calculated. The optimal cut-off of major axis length for predicting mesorectal LN metastasis was calculated to be 3 mm. When cut-off size of the LN was set at 2 mm, sensitivity and specificity for positive detection were calculated as 0.88 and 0.27, respectively. At a cut-off of 4 mm, sensitivity was 0.52 and specificity was 0.83. (Fig 3). When the cut-off was 8 mm, as the value used clinically for positive diagnosis of rectal adenocarcinoma was adapted to this study, LN metastasis was diagnosed in only 7 patients (21%) on preoperative CT. According to these results, the optimal cut-off was considered to be 3 mm, and we diagnosed LN metastasis in 27 patients (82%) on preoperative CT, but sensitivity and specificity for positive detection at this size were calculated as 0.82 and 0.49, respectively Table 2.

**Table 2:** Reliability of predicting lymph node metastasis on CT at each cut-off vale.

Cut-off value	CT	pN(+)	pN(-)	Total	Sensitivity	Specificity
8 mm	≧8mm	7	0	7		
	<8mm	26	69	95	0.21	1
3 mm	≧3mm	27	35	62		
	<3mm	6	34	40	0.82	0.49

### Long-term outcomes

Median follow-up in this series was 37 months (range, 0.7–113 months). One patient (1%) developed local recurrence and two patients (2%) developed distant recurrences among the metastasis-positive LN group. Three patients died, with two deaths related to multiple liver metastases from the primary tumor and one death because of primary lung cancer. The 3-year relapse-free survival rate after excluding clinical stage IV cases (n = 2) was 96%.

## Discussion

This study investigated the relationship between LN size and LN metastasis from rectal NET in patients who underwent TME or TSME. This study performed CT with 5 mm cuts, representing a common modality in clinical practice to evaluate metastatic lesions secondary to rectal NETs. Although MRI is well known to produce better images to assess the circumferential margin and detect existence of LNs than CT, in this study MRI would not contribute to predict metastasis, because many patients showed no findings on CT. We thus did not perform MRI in many cases. Another imaging tool, octreotide scan, is useful for determining metastatic diseases in general, although higher-grade colorectal NET lesions are often missed [11] and previous studies have reported CT and MRI as superior to octreotide scan for detecting metastasis [19].

Our results showed metastatic LNs were significantly larger than non-metastatic LNs. However, the long axis of most metastatic LNs was too small to predict positive LN from preoperative CT. Although several studies of LN size with colorectal adenocarcinoma have been reported [20–24], few studies have investigated the clinical value of LN size with rectal NET. Studies relating to adenocarcinoma have reported mean size of non-metastatic /metastatic LNs as 4.2 mm /6 mm [20], 2.94 mm/4.59 mm [21], and 3.4 mm/6.0 mm [23], with 69% and 45% of metastatic LNs <5 mm in diameter, respectively [20,21]. Our study showed that in the mesorectum, mean sizes for non-metastatic/metastatic LNs were 2.39/4.36 mm and 81% of positive LNs were <5 mm in diameter, implying a smaller size of metastatic LNs for rectal NET compared to that of rectal adenocarcinoma. While the sensitivity of preoperative CT for detecting metastasis positive LN was 0.45–0.73 in colorectal adenocarcinoma [25,26], the sensitivity of CT in rectal NET in our series was a rather lower 0.21. Kim et al. [19] speculated that the differences between metastatic LN of NET and adenocarcinoma of the rectum were attributable to the absence of stromal reactions and macroscopic extracapsular extension. They suggested that these features make metastatic LN difficult to diagnose and lead to small metastases being missed in histopathological diagnosis [19]. No reports, however, have provided reasons for smaller metastatic LN of rectal NET than those of adenocarcinoma. We wonder if this disease is derived from the Kulchitsky cells located in the crypts of Lieberkuhnt [27], originating in the mucosal layer near the submucosa and potentially showing some unique characteristics likely to result in metastasis even with a small primary tumor. These topics remain for future investigations.

Our study showed that 80% of the positive LNs were smaller than 5 mm. The 5-mm interval generally used in CT examinations would thus detect obviously swollen LNs, but may miss small LN metastases and thus increase the difficulty of preoperative diagnosis. In our study, ER/TAR was performed before the operation in most cases (n = 68, 66%). Hence, the LNs may have increased in size after the primary endoscopic procedure due to secondary inflammatory changes. However, the mean sizes of LNs were almost the same and no significant differences were seen between ER/TAR and no-ER/TAR groups. This could indicate that ER/TR induced only slight inflammatory changes in LNs in our study patients because of the time interval between endoscopic resection and operation, and because of the small size of tumors.

As far as distribution of positive LNs in rectal NET in our series was concerned, most positive LNs were located along the superior rectal artery, implying that dissection around the origin of the inferior mesenteric artery may be unnecessary.

In terms of dissection of LPLNs in rectal NET, no evidence-based data have been published to date. In our institution, selective LPLD has been performed for patients with rectal cancer and swollen LNs >7 mm in longitudinal diameter [18]. This indication for LPLD was adapted to rectal NET, with the results interestingly but accurately showing a positive rate of 86% (6/7 patients). Moreover, 4 of the 6 patients (66%) who had positive LPLNs showed no LN metastases in the mesorectum, implying that some patients may first experience first lymphatic flow to this region, as with rectal cancer. We performed curative resection with selective LPLD for 4 patients (66%), but the remaining 2 patients developed recurrence. On the other hand, most patients did not undergo selective LPLD, and no patients developed LPLN recurrence, but this may have been a result of the small sample size. The difference of the size of the positive LNs between mesorectum and lateral region is attributed to the difference of indication for dissection to these two areas. Mesorectal dissection with rectal resection is performed based on tumor size, depth of invasion, and histological findings whereas lateral lymph node dissection is performed based on the size of the lateral lymph node itself. It, however, would not be necessary to adopt the same cut off size of lymph node in the mesorectum to the lateral region because of no lateral lymph node recurrences in even cases without lateral lymph node dissection. Considering the poor prognosis of patients who develop LN metastasis in the lateral pelvic region in cases of rectal cancer, performing LPLD might be effective for rectal NET patients despite the shortage of cases. An important issue to address in the future is suitable surgical treatment for rectal NET in terms of not only TME/TSME, but also LPLD.

Some limitations need to be considered when interpreting the results of this study. First, this study was a single-center, retrospective analysis. Second, a section thickness of 5mm on MDCT was used for preoperative examinations to determine LN metastasis, and may have made smaller LNs (such as those <3 mm in size) difficult to detect. Third, a definite standardized protocol using methods like the methylene blue or fat clearance method [24] for dissecting and measuring LNs was not applied. Fourth, the size of pathologically preserved tissue may be different from that imaged by CT. Kono et al. [23] reported that fresh LNs in rectal cancer shrunk by about 30% after formalin fixation. Hence, the LN size in our study, which was measured in pathological preserved tissue, may be smaller than the size of LNs on CT.

## Conclusions

This study achieved relatively favorable long-term outcomes, implying a reasonable treatment strategy in our institution and showed the difficulty of predicting LN metastasis on CT. Now that even quite small NET is often detected on screening colonoscopy[28], pathological findings if resected by endoscopy and the size over 1cm were properly important factors to predict LN metastasis, rather than preoperative CT imaging.

## Supporting information

S1 Table Clinicopathological characteristics. (XLSX)

S2 Table Long-axis diameter of retrieved 1169 mesorectal LNs. (XLSX)

S3 Table The location of metastatic LNs. (XLSX)

S4 Table Lateral pelvic lymph nodes. (XLSX)

## References

[1] FT B. World Health Organization. International Agency for Research on Cancer.(2010 4th edition) WHO classification of tumors of the digestive system. 2010.

[2] McConnell YJ. Surgical management of rectal carcinoids: trends and outcomes from the Surveillance, Epidemiology, and End Results database (1988 to 2012). Am J Surg. 2016;211(5):877–85. Epub 2016/04/07. 10.1016/j.amjsurg.2016.01.008 . 27048945

[3] Konishi T, Watanabe T, Kishimoto J, Kotake K, Muto T, Nagawa H, et al Prognosis and risk factors of metastasis in colorectal carcinoids: results of a nationwide registry over 15 years. Gut. 2007;56(6):863–8. Epub 2007/01/11. 10.1136/gut.2006.109157 17213340 PMC1954860

[4] Soga J. Carcinoids and their variant endocrinomas. An analysis of 11842 reported cases. J Exp Clin Cancer Res. 2003;22(4):517–30. . 15053292

[5] Takatsu Y, Fukunaga Y, Nagasaki T, Akiyoshi T, Konishi T, Fujimoto Y, et al Short- and Long-term Outcomes of Laparoscopic Total Mesenteric Excision for Neuroendocrine Tumors of the Rectum. Dis Colon Rectum. 2017;60(3):284–9. Epub 2017/02/09. 10.1097/DCR.0000000000000745 . 28177990

[6] Yao JC, Hassan M, Phan A, Dagohoy C, Leary C, Mares JE, et al One hundred years after "carcinoid": epidemiology of and prognostic factors for neuroendocrine tumors in 35,825 cases in the United States. J Clin Oncol. 2008;26(18):3063–72. Epub 2008/06/21. 10.1200/JCO.2007.15.4377 . 18565894

[7] Bajetta E, Ferrari L, Procopio G, Catena L, Ferrario E, Martinetti A, et al Efficacy of a chemotherapy combination for the treatment of metastatic neuroendocrine tumours. Ann Oncol. 2002;13(4):614–21. Epub 2002/06/12. . 12056713 10.1093/annonc/mdf064

[8] Bajetta E, Procopio G, Ferrari L, Catena L, Del Vecchio M, Bombardieri E. Update on the treatment of neuroendocrine tumors. Expert Rev Anticancer Ther. 2003;3(5):631–42. Epub 2003/11/06. 10.1586/14737140.3.5.631 . 14599087

[9] Niederhuber JE, Fojo T. Treatment of metastatic disease in patients with neuroendocrine tumors. Surg Oncol Clin N Am. 2006;15(3):511–33, viii. Epub 2006/08/03. 10.1016/j.soc.2006.05.004 . 16882495

[10] Kulke MH, Shah MH, Benson AB 3rd, Bergsland E, Berlin JD, Blaszkowsky LS, et al Neuroendocrine tumors, version 1.2015. J Natl Compr Canc Netw. 2015;13(1):78–108. Epub 2015/01/15. . 25583772 10.6004/jnccn.2015.0011

[11] Caplin M, Sundin A, Nillson O, Baum RP, Klose KJ, Kelestimur F, et al ENETS Consensus Guidelines for the management of patients with digestive neuroendocrine neoplasms: colorectal neuroendocrine neoplasms. Neuroendocrinology. 2012;95(2):88–97. Epub 2012/01/21. 10.1159/000335594 . 22261972

[12] de Mestier L, Brixi H, Gincul R, Ponchon T, Cadiot G. Updating the management of patients with rectal neuroendocrine tumors. Endoscopy. 2013;45(12):1039–46. Epub 2013/10/29. 10.1055/s-0033-1344794 . 24163193

[13] Anthony LB, Strosberg JR, Klimstra DS, Maples WJ, O'Dorisio TM, Warner RR, et al The NANETS consensus guidelines for the diagnosis and management of gastrointestinal neuroendocrine tumors (nets): well-differentiated nets of the distal colon and rectum. Pancreas. 2010;39(6):767–74. Epub 2010/07/29. 10.1097/MPA.0b013e3181ec1261 . 20664474

[14] Zhou X, Xie H, Xie L, Li J, Fu W. Factors associated with lymph node metastasis in radically resected rectal carcinoids: a systematic review and meta-analysis. J Gastrointest Surg. 2013;17(9):1689–97. Epub 2013/07/03. 10.1007/s11605-013-2249-7 . 23818123

[15] Shields CJ, Tiret E, Winter DC, International Rectal Carcinoid Study G. Carcinoid tumors of the rectum: a multi-institutional international collaboration. Ann Surg. 2010;252(5):750–5. Epub 2010/11/03. 10.1097/SLA.0b013e3181fb8df6 . 21037430

[16] Lowry AC, Simmang CL, Boulos P, Farmer KC, Finan PJ, Hyman N, et al Consensus statement of definitions for anorectal physiology and rectal cancer: report of the Tripartite Consensus Conference on Definitions for Anorectal Physiology and Rectal Cancer, Washington, D.C., May 1, 1999. Dis Colon Rectum. 2001;44(7):915–9. Epub 2001/08/10. . 11496067 10.1007/BF02235475

[17] Kasuga A, Chino A, Uragami N, Kishihara T, Igarashi M, Fujita R, et al Treatment strategy for rectal carcinoids: a clinicopathological analysis of 229 cases at a single cancer institution. J Gastroenterol Hepatol. 2012;27(12):1801–7. Epub 2012/06/30. 10.1111/j.1440-1746.2012.07218.x . 22743039

[18] Akiyoshi T, Ueno M, Matsueda K, Konishi T, Fujimoto Y, Nagayama S, et al Selective lateral pelvic lymph node dissection in patients with advanced low rectal cancer treated with preoperative chemoradiotherapy based on pretreatment imaging. Ann Surg Oncol. 2014;21(1):189–96. Epub 2013/08/22. 10.1245/s10434-013-3216-y . 23963871

[19] Kim BC, Kim YE, Chang HJ, Lee SH, Youk EG, Lee DS, et al Lymph node size is not a reliable criterion for predicting nodal metastasis in rectal neuroendocrine tumours. Colorectal Dis. 2016;18(7):O243–51. Epub 2016/05/12. 10.1111/codi.13377 . 27166857

[20] Cserni G. The influence of nodal size on the staging of colorectal carcinomas. J Clin Pathol. 2002;55(5):386–90. Epub 2002/05/03. 11986347 10.1136/jcp.55.5.386 PMC1769647

[21] Rodriguez-Bigas MA, Maamoun S, Weber TK, Penetrante RB, Blumenson LE, Petrelli NJ. Clinical significance of colorectal cancer: metastases in lymph nodes < 5 mm in size. Ann Surg Oncol. 1996;3(2):124–30. Epub 1996/03/01. . 8646511 10.1007/BF02305790

[22] Langman G, Patel A, Bowley DM. Size and distribution of lymph nodes in rectal cancer resection specimens. Dis Colon Rectum. 2015;58(4):406–14. Epub 2015/03/10. 10.1097/DCR.0000000000000321 . 25751797

[23] Kono Y, Togashi K, Utano K, Horie H, Miyakura Y, Fukushima N, et al Lymph Node Size Alone Is Not an Accurate Predictor of Metastases in Rectal Cancer: A Node-for-Node Comparative Study of Specimens and Histology. Am Surg. 2015;81(12):1263–71. Epub 2016/01/07. . 26736166

[24] Markl B, Rossle J, Arnholdt HM, Schaller T, Krammer I, Cacchi C, et al The clinical significance of lymph node size in colon cancer. Mod Pathol. 2012;25(10):1413–22. Epub 2012/06/12. 10.1038/modpathol.2012.92 . 22684222

[25] Fisher KS, Zamboni WA, Ross DS. The efficacy of preoperative computed tomography in patients with colorectal carcinoma. Am Surg. 1990;56(6):339–42. Epub 1990/06/01. . 2350106

[26] Kobayashi H, Kikuchi A, Okazaki S, Ishiguro M, Ishikawa T, Iida S, et al Diagnostic performance of multidetector row computed tomography for assessment of lymph node metastasis in patients with distal rectal cancer. Ann Surg Oncol. 2015;22(1):203–8. Epub 2014/08/16. 10.1245/s10434-014-3972-3 . 25124470

[27] Eggenberger JC. Carcinoid and other neuroendocrine tumors of the colon and rectum. Clin Colon Rectal Surg. 2011;24(3):129–34. Epub 2012/09/04. 10.1055/s-0031-1285996 22942794 PMC3311499

[28] Taghavi S, Jayarajan SN, Powers BD, Davey A, Willis AI. Examining rectal carcinoids in the era of screening colonoscopy: a surveillance, epidemiology, and end results analysis. Dis Colon Rectum. 2013;56(8):952–9. Epub 2013/07/11. 10.1097/DCR.0b013e318291f512 . 23838863

